# Scientific Items

**Published:** 1882-09-20

**Authors:** 


### SCIENTIFIC ITEMS.

**Canals on the Planet Mars.**—The Rev. T. W. Webb, author of "Celestial Objects for Common Telescopes," writes thus to the London Times respecting Schiaparelli's discovery of "canals" on the planet Mars: "It has long been known that the planet Mars is so mapped out into brighter and darker portions as to suggest the idea of continents and oceans, and the analogy thus implied with the arrangements of our own globe is strengthened by the existence of brilliant white patches, as of snow or ice, situated at or near the planet's poles of rotation, and varying in extent with its changing seasons, as well as by occasional differences in outline or coloring, which may well be explained by the supposition of a vaporous atmosphere.

"In the autumn of 1877 and spring of 1878, a number of minute, straight black or dusky bands were detected by Schiaparelli, traversing and subdividing the supposed continents in various directions. These have been called, from their aspects, "canals," though, of course, their scale entitles them rather to the appellation of straits, or very long, narrow arms of the sea. A view of these had previously been seen by various observers, but to the Italian astronomer belonged the credit of developing and delineating them as a system. At the ensuing return of the planet in 1879-80, they were again detected and drawn by him with very little difference. But during the course of last January and February he has been so fortunate as to perceive the duplication of these dark streaks by the addition of parallel lines of similar character and length in no fewer than twenty instances, covering the equatorial region with a strange and mysterious network, to which there is nothing even remotely analogous on the earth, and which leads us at once to see how premature have been our conclusions in this respect, and how far we still are from any adequate conception of the real constitution even of our nearest neighbor but one in the solar system."

The results of the tenth census are given to the public in installments, the last of which, it is hoped, may be reached before the eleventh census is begun. Among other facts which have thus far been divulged are the following: The number of families in the United States is 9,945,916; average number of persons to the family, 5.04; number of dwellings, 8,955,812; average number of persons to a dwelling, 5.60. The average number of persons to the square mile is 13.92, but the distribution is highly unequal, the District of Columbia having 2,537, and Rhode Island 221 persons to the square mile, while in Wyoming Territory one person is distributed over five square miles. If the whole area of the country were divided pro rata among the inhabitants, each individual would possess 45.98 acres, or each family 231.74 acres: a clear indication that the period of overcrowding on the American continent is still very comfortably remote.—*Mech'l News.*

**The "Smartness" of Worms.**—"I have made some of my most interesting studies of nature in the morning," said Seth Green. "That is the time to see the insects at their best—to see the mud wasps stinging the spiders without killing them, and packing them away where they are kept alive for weeks to be used when needed. I have seen a small green worm hanging down on a web. An ant, stationed on the limb above, pulls up the web, and, just as the worm comes within reach of his tiny claws, down drops Mr. Worm. The ant pulls up again and again and the worm lets out another reef and goes down. This sort of thing continues until finally the ant grapples the worm and both go down together in a grand scramble, in which the worm manages to shake off the ant. This leaves the worm on the ground. His web is so strong that the other end is still fastened to the limb above. What does Mr. Ant do? Give it up? No, sir. I have seen him go up the trunk of that tree, crawl out onto the same limb and go to work again pulling up the same web. Then, after another battle, I have known the ant to get the better of the fight and lug the worm off to his hole, three rods away.—*Ex.*

In round numbers, there are now 100,000 miles of railroad in the United States. The length of lines of which the track has been laid exceeds this figure somewhat; the number of miles in operation may fall a trifle below it. But the close of the year 1882 will see more than 100,000 miles of railway in running condition, as the lines are stretching out at the rate of 200 miles per week. The cost of this 100,000 miles of road, first and last, has been about $6,000,000,000; and Henry V. Poor, the special historian of railway progress in America, believes that ten years hence a total of 200,000 miles will have been reached, representing an investment $10,000,000,000.

**Greatness.**—Nothing seems to me clearer than that greatness, whether mental or moral, depends mainly on innate powers and impulses which are in part inherited, but by no means wholly so. There is only this difference, that the highest goodness may ultimately be reached by whoever will seek it steadily and passionately; but no strength or persistency of desire for intellectual distinction can possibly achieve it, unless the brain is favorably formed. It depends mainly on natural disposition whether we choose to be saintly; but, if we do choose, then saints we shall become. We must be born kings of thought, or we shall never wear that crown.—*Ex.*

**Cleans Pens.**—A writer in a German paper states that it is a custom in offices in that country to have a sliced potato on the desk in commercial houses. He does not state whether the esculent should be raw or not, but as it is not for the purpose of making kartoffel-salat, it is probably raw. It is used to clean steel pens, and generally act as a pen-wiper. It removes all ink-crust, and gives a peculiarly smooth flow to the ink. He also states that the Hamburg clerks pass new pens two or three times through a gas flame, and then the ink will flow freely.

